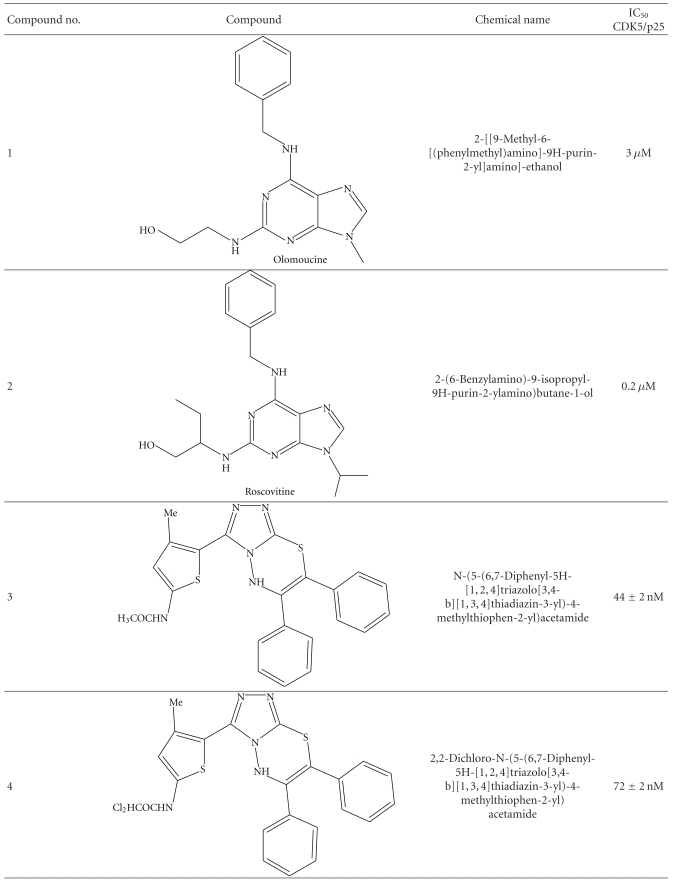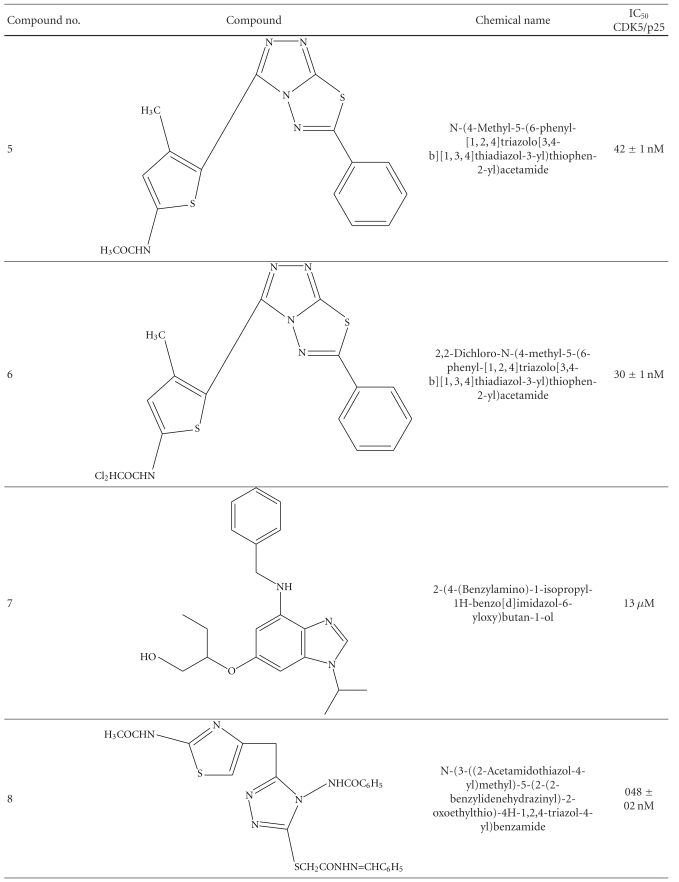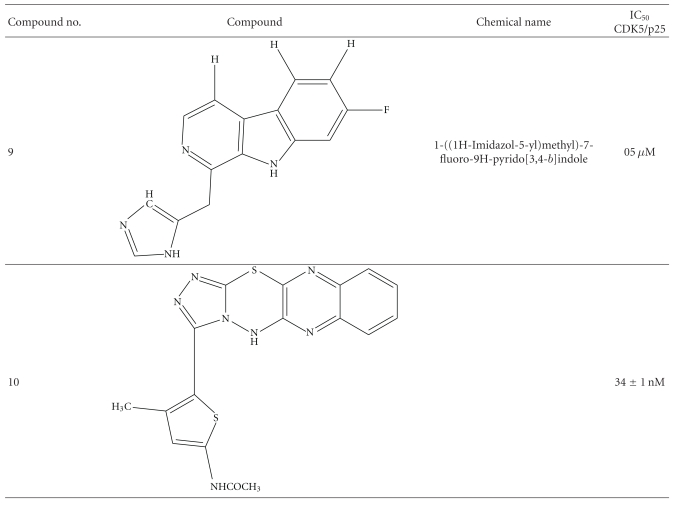# Cyclin-Dependent Kinase 5/p35/p39: A Novel and Imminent Therapeutic Target for Diabetes Mellitus

**DOI:** 10.1155/2011/530274

**Published:** 2011-10-19

**Authors:** Danish Ahmed, Manju Sharma

**Affiliations:** ^1^Department of Pharmaceutical Sciences, Faculty of Health, Medical Sciences, Indigenous and Alternative Systems of Medicine, Sam Higginbottom Institute of Agriculture, Technology & Sciences (SHIATS), Allahabad 211007, India; ^2^Department of Pharmacology, Faculty of Pharmacy, Jamia Hamdard, New Delhi 110062, India

## Abstract

Present therapies to minify hyperglycaemia and insulin resistance mainly target ATP-sensitive K^+^ channels (K_ATP_) of pancreatic cells and PPAR-**γ** to enhance the insulin secretion and potential for GLUT expression, respectively. These current approaches are frequently associated with the various side effects such as hypoglycaemia and cardiovascular adverse events. CDK5 is a serine/threonine protein kinase, which forms active complexes with p35 or p39 found principally in neurons and in pancreatic **β** cells. Pieces of evidence from recent studies recommend the vital role of CDK5 in physiological functions in nonneuronal cells such as glucose-stimulated insulin secretion in pancreatic cells. Inhibition of CDK5 averts the decrease of insulin gene expression through the inhibition of nuclear translocation of PDX-1 which is a transcription factor for the insulin gene. The present pieces of evidence designate that CDK5 might be a potential drug target for the regulation of glucose-stimulated insulin secretion in the treatment of diabetes mellitus.

## 1. Introduction

Cyclin-dependent kinases (CDKs) play essential roles in the regulation of cell division cycle [2]. Cyclin-dependent kinases (CDKs) represent key molecules involved in regulation of the cell cycle. CDKs are serine/threonine kinases that become active only when associated with a regulatory partner (e.g., cyclins or other proteins). CDK/cyclin holoenzymes are activated by phosphorylation, which is catalyzed by CDK-activator kinase (CAK).

CDK5 is a serine-threonine kinase that is ubiquitously expressed in mammalian tissues [3]. But the kinase activity is limited in neurons because of the predominant expression of its activators, p35 and p39, in neurons [3–6]. Recent studies have shown that p35 and p39 are expressed in pancreatic beta cells [7, 8] suggesting the possible activation and potential role of CDK5 in insulin secretion.

Excellent recent researches have also documented the high levels of CDK5 activity and p35 expression in both pancreatic beta cells and beta-cell-derived cell lines [9]. Studies also suggested that two different pathways are mainly responsible for stimulating insulin secretion: a triggering pathway, in which depolarization by closure of the K^+^ATP channel directly activates L-VDCC and results in the rise of cytosolic Ca^2+^, and an augmentative pathway, in which cAMP is an important mediator [10].

The regulation of CDK5 kinase activity is somewhat different from that of other CDKs. It is well established that phosphorylation of Thr160 within CDK2 by CAK and dephosphorylation of Tyr15 by cdc25 are necessary for the maximum activation [11, 12]. Although there are contradictory results regarding the effect of tyrosine phosphorylation on CDK5 activity, it seems that tyrosine-dependent regulation is significant for CDK5 [13, 14]. At present, it is generally thought that binding of p35 or p39 to CDK5 is both necessary and sufficient to activate CDK5 kinase. Type 2 diabetes is characterized by a deficit in b-cell mass with increased beta-cell apoptosis as well as a deficit in b-cell function [15]. 

Neurons in Alzheimer's disease and *β*-cells in type 2 diabetes are characterized by endoplasmic reticulum (ER) stress-induced apoptosis associated with formation of misfolded toxic oligomers of locally expressed amyloidogenic proteins (Alzheimer's b-protein [IAbP] in brain and islet amyloid polypeptide [IAPP] in beta-cells) [16, 17].

## 2. Cyclin-Dependent Kinase 5 and Glucotoxicity

Glucose enhances p35 gene expression, promoting the formation of active p35/CDK5 complexes that in turn regulate the expression of the insulin gene [7]. Transient elevations of extracellular glucose promote pancreatic beta-cell function and survival [3, 4] whereas chronic elevations of glucose have the opposite effect, impairing beta-cell function and survival [18–20]. The deleterious effects of chronically elevated glucose are referred to as glucotoxicity.

The suppression of insulin gene expression by glucotoxicity in beta cells involves several transcription factors and different pathophysiological mechanisms. These mechanisms include the translocation of PDX-1 (also known as STF-1, IPF-1, and IDX-1) from the nucleus to the cytoplasm [21]. 

**Figure 1:**
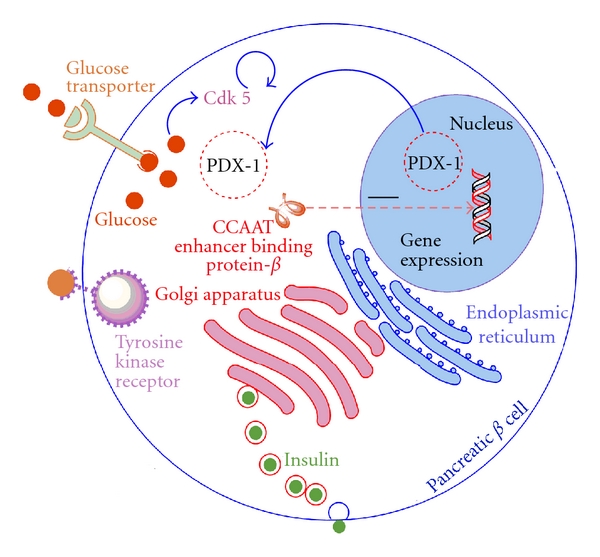
Regulation of pancreatic *β*-cell function by Cyclin-Dependent Kinase 5 (CDK5). Cyclin-Dependent Kinase 5 (CDK5) is overactivated by high glucose or by signal downstream of tyrosine kinase receptor. The PDX 1 is translocated from nucleus to cytoplasm due to activation of CDK5. That results in the suppression of gene expression by induction of transcription factors such as CCAAT enhancer binding protein *β*, which acts as negative regulator of insulin gene transcription.

There are pieces of evidence which strongly suggest that overstimulation of CDK5 is involved in the cytoplasmic translocation of PDX-1 during glucotoxicity. Recent works indicates that elevation of CDK5 activity is due to an increased expression level of its p35 activator without changes in the level of CDK5 protein. The overall effects of increased activity of CDK5 are decreased rates of insulin release, reduced insulin production and diminished insulin gene expression (see Figure 1) [22, 23]. 

## 3. Regulation of Insulin Release by CDK 5

**Figure 2:**
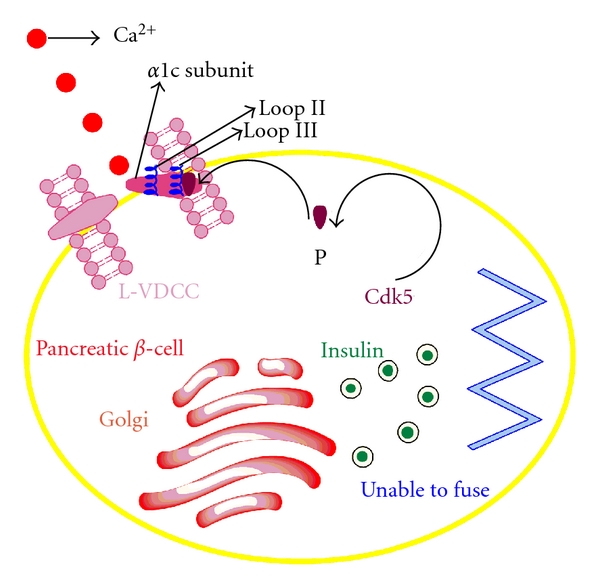
Regulation of insulin secretion by CDK5: cyclin-dependent kinase 5 (CDK5) prevents the secretion of insulin by phosphorylating the loop II and loop III of *α*1c subunit of L-type voltage-dependent calcium channels (L-VDCCs) so that calcium could not find the entry into the pancreatic *β* cell as the L-VDCC channel activity is deterred due to the phosphorylation leading to the decreased concentration of cytosolic Ca^2+^.

Similar to other cyclin-dependent kinases, CDK5 too needs cyclin proteins to activate the kinase activity, which have been recognized as p35 and its isoform p39 (see Figure 2) [3–5].

It has been shown that CDK5 is associated with exocytosis machinery and is also involved in the neurotransmitter release. As the neuron and pancreatic *β*-cell allocated to the same secretion mechanism, it is obvious to understand that CDK5 would adjust the insulin secretion in pancreatic *β* cells. Insulin secretion is about to begin when calcium is influxed through the L-VDCC in reaction to enhanced level of extracellular glucose. CDK5 phosphorylates loop II-III of the *α*1c subunit of L-VDCC and restrains the channel activity that ultimately results in the inhibition of glucose-stimulated insulin secretion.

## 4. Role of CDK5 in Regulation of PPAR-*γ* Activity

**Figure 3:**
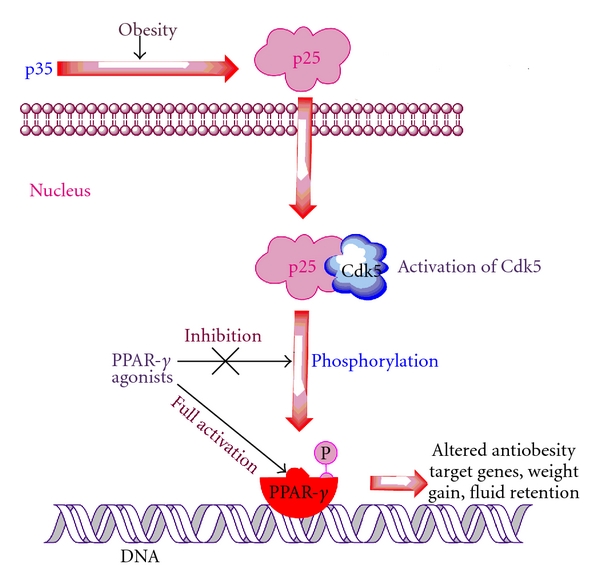
Choi et al. [1] show that PPAR*γ* activity is controlled by the CDK5. Obesity leads to the various signals that cause the cleavage of p35 to p25 which will then translocates to the nucleus and forms a bond with CDK5 and activates it. CDK5 phosphorylates the PPAR*γ* receptor on serine residue 273 averts the transcription of antiobesity effects, while the full activation of PPAR*γ* by PPAR*γ* agonists may probably responsible for the weight gain and Fluid retention.

A Recent study clearly demonstrated that CDK5 regulates the PPAR-*γ* activity in the pancreatic *β* cells [1]. In their findings they make obvious that the enzyme cyclin-dependent kinase 5 (CDK5) phosphorylates PPAR*γ* on serine residue 273. Activation of CDK5 itself involves truncation of the p35 protein to p25, possibly in response to cytokines or other proinflammatory signals p25 then translocates to the nucleus, where it associates with, and activates, CDK5 in a way that is evocative of the activation of other CDK enzymes. Phosphorylation of CDK5 causes the alteration and inhibition of specific antiobesity target genes (Figure 3) [1]. Enigmatically, the antidiabetic PPAR*γ* ligands that were previously considered to act solely by activating PPAR*γ* potently inhibit its CDK5-mediated phosphorylation [1], probably by inducing a conformational change in PPAR*γ*.

The data from the above study demonstrates that antidiabetic PPAR*γ* ligands inhibit CDK5 phosphorylation of PPAR*γ* in vivo and reverse changes in gene expression linked to this modification. Treatment with roscovitine, a CDK5 inhibitor, significantly suppressed CDK5-mediated phosphorylation and most of the gene set regulated by the phosphorylation of PPAR*γ*. It is important in the research study that the therapeutic doses used in animals did not lead to a complete inhibition of the CDK5 phosphorylation of PPAR*γ*. It is clear from the vital research that CDK5 plays an important role in adipogenic diabetes and the inhibition of CDK5-mediated phosphorylation of PPAR*γ* receptor could lead to the improvement in the serious side effects associated with the PPAR*γ* agonists which may occur through their classical agonist actions. Therefore, the full PPAR*γ* ligands activate the PPAR*γ* receptor which could be the reason for weight gain and fluid retention. We need to more robust therapy which could only target the phosphorylation of PPAR*γ*. 

## 5. CDK5 Inhibitors as Potential Therapeutic Target

Cyclin-dependent kinases (CDKs) play indispensable roles in the regulation of cell division cycle [24]. There are many pieces of evidence which have shown the importance of CDKs in the diabetes [1]. The initial search for CDK inhibitors was commenced because of their antitumor potential and was mostly based on the use of CDK1/cyclin B as a molecular target. Frequent deregulations of CDK in cancers provided the main thrust to the vigorous rummage around for pharmacological inhibitors of these kinases. 

CDK5 is a serine/threonine kinase ubiquitously expressed in the mammalian tissues, with best characterized functional role in the Alzheimer's disease [25]. Although the origin of pancreatic endocrine cells is endodermal but it shares many molecular and cellular characteristics with neural ectoderm-derived neurons. Because of the similarities between neurons and pancreatic *β* cells and between the neural degeneration of Alzheimer's disease and the deterioration of pancreatic *β*-cell functioning, CDK5 plays an important role in the pathogenesis of diabetes mellitus [7]. It is now clear that phosphorylation of serine and threonine residues plays a fundamental role in pathogenesis of adipogenic diabetes and constitutes a major pharmacological target [1]. CDK5 has trifling enzymatic activity and necessitates association with regulatory proteins for comprehensive activation. Two noncyclic proteins, p35 and p39, have been discovered as CDK5 activators that are restricted in the cell membrane.

Frequent deregulations of CDKs in cancer [26] provided the main drive to the active search for pharmacological inhibitors of CDK5. The first pharmacological CDK inhibitors (6-dimethylaminopurine and isopentenyladenine) were neither active nor selective. However the discovery paved the way for the synthesis of many other CDK inhibitors and proved to be the starting point for further research. More than 50 inhibitors for CDKs have now been reported.

Despite significant chemical assortment, all CDK inhibitors share some common properties: (1) they are flat, (2) they combine mostly by hydrophobic interactions, (3) they compete with ATP for binding, (4) they have low molecular weights.

**Scheme 1:**
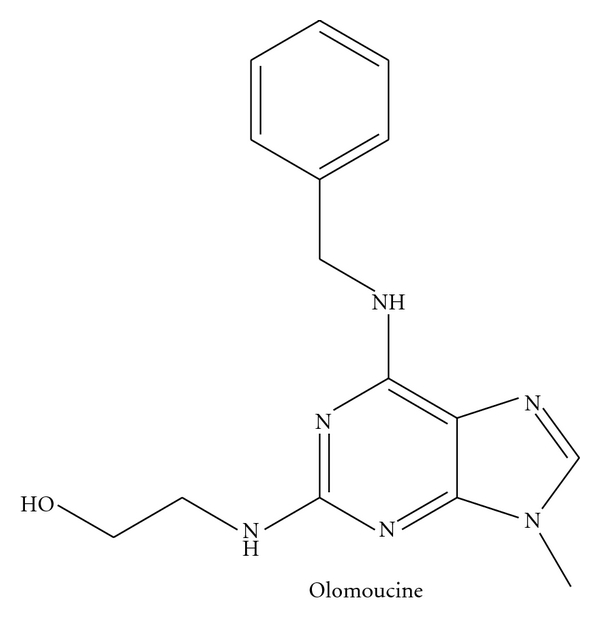


**Scheme 2:**
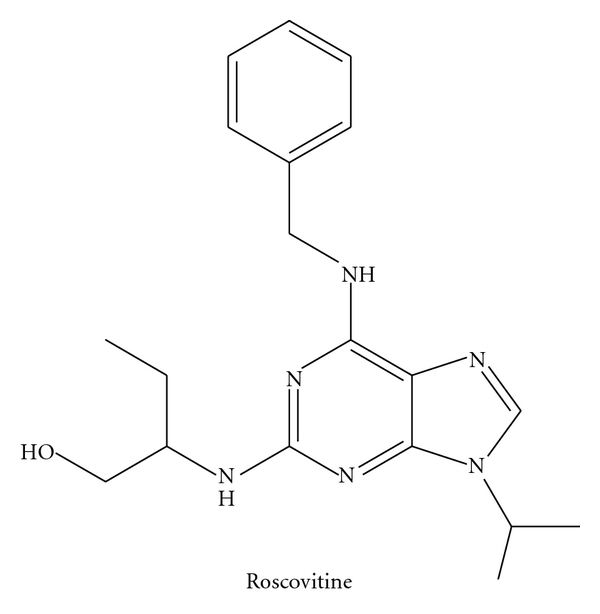


Hitherto two types of CDK5 inhibitor, chemical compounds, and peptides have been acknowledged. The most recurrently utilized chemical compounds are purine analogues such as Roscovitine and Olomoucine. These inhibitors compete with ATP for binding to CDK5 and form hydrogen bonds with CDK5. However due to high homology of the primary amino acid sequence and the 3D structure of CDK5 and other CDKs, particularly in the kinase domain, roscovitine and olomoucine can also inhibit other CDKs to some extent (see Schemes  1 and 2).

To surmount this problem, CDK5 inhibitory peptides have been discovered. These peptides are obtained from an explicit region in p35 protein and are highly specific for CDK5. Studies using such CDK5 inhibitory peptides suggest that these peptides could prevent CDK5-mediated cell death. However, the IC50s of these peptides are higher and the membrane permeability of these peptides is lower than those of roscovitine and olomoucine.

## 6. Novel CDK5/p25 Inhibitors

Recently there were comprehensive researches done to target specifically the CDK5/p25. Researchers have found a range of compounds with high potency. Some of them are described here with IC_50_ in nM (see Table 1).

## 7. Docking Studies of Some Novel CDK5 Inhibitors

In order to understand the structural requirements of the inhibitors with respect to their bioactivity profile enhancements against cyclin-dependent kinase 5/p25 (CDK5/p25), molecular docking with Discovery Studio Client v2.5.0.9164 (Accelrys) was carried out.

**Figure 4:**
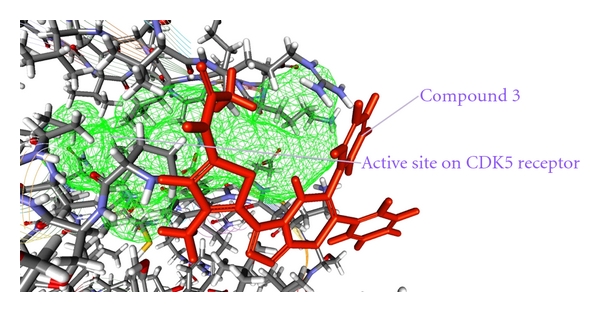
The docked conformation of compound 3 with the receptor CDK5/p25 (PDB: 3O0G). Compound 3 is indicated by red color while the most active site of the CDK5 receptor is shown with green color.

**Figure 5:**
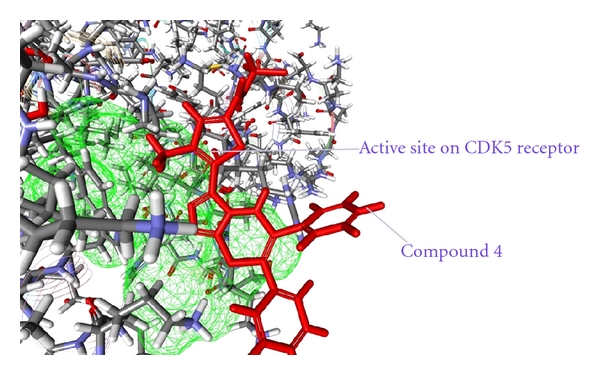
The docked conformation of compound 4 with the receptor CDK5/p25 (PDB: 3O0G). Compound 4 is indicated by red color while the most active site of the CDK5 receptor is shown with green color.

**Figure 6:**
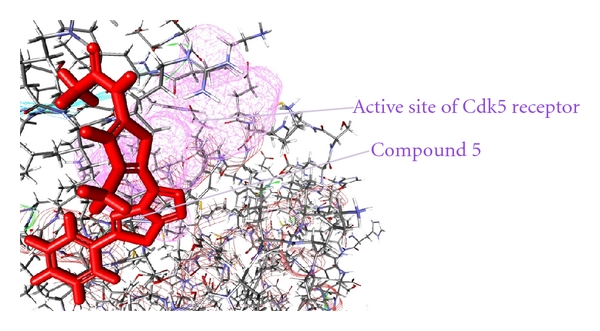
The docked conformation of compound 5 with the receptor CDK5/p25 (PDB: 3O0G). Compound 5 is indicated by red color while the most active site of the CDK5 receptor is shown with purple color.

**Figure 7:**
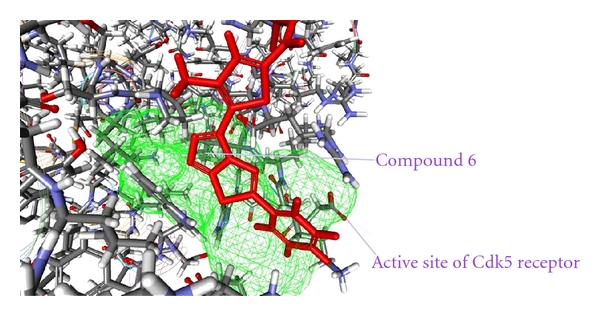
The docked conformation of compound 6 with the receptor CDK5/p25 (PDB: 3O0G). Compound 6 is indicated by red color while the most active site of the CDK5 receptor is shown with green color.

**Figure 8:**
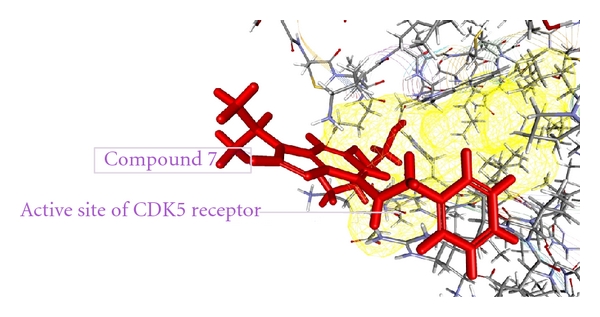
The docked conformation of compound 7 with the receptor CDK5/p25 (PDB: 3O0G). Compound 7 is indicated by red color while the most active site of the CDK5 receptor is shown with yellow color.

**Figure 9:**
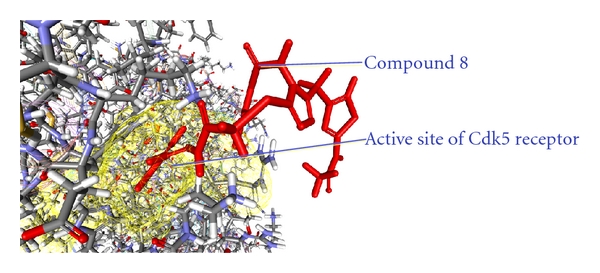
The docked conformation of compound 8 with the receptor CDK5/p25 (PDB: 3O0G). The compound 8 is indicated by red color while the most active site of the CDK5 receptor is shown with yellow color.

**Figure 10:**
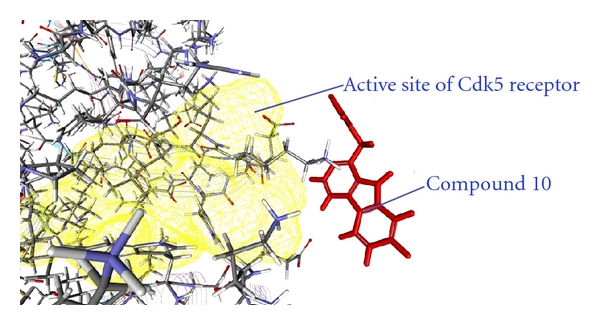
The docked conformation of compound 10 with the receptor CDK5/p25 (PDB: 3O0G). Compound 10 is indicated by red color while the most active site of the CDK5 receptor is shown with yellow color.

**Figure 11:**
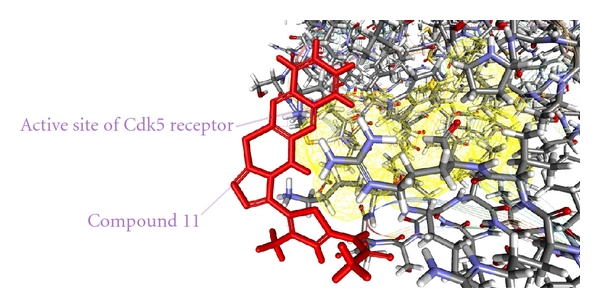
The docked conformation of compound 11 with the receptor CDK5/p25 (PDB: 3O0G). Compound 11 is indicated by red color while the most active site of the CDK5 receptor is shown with yellow color.

**Figure 12:**
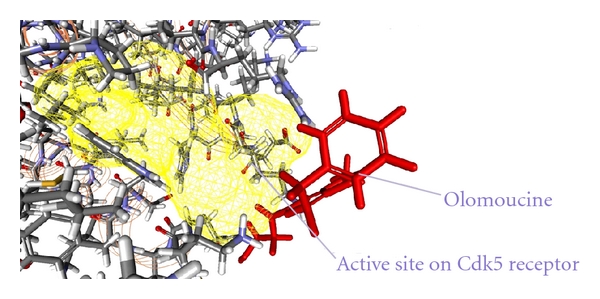
The docked conformation of Olomoucine with the receptor CDK5/p25 (PDB: 3O0G). The Olomoucine is indicated by red color while the most active site of the CDK5 receptor is shown with yellow color.

**Figure 13:**
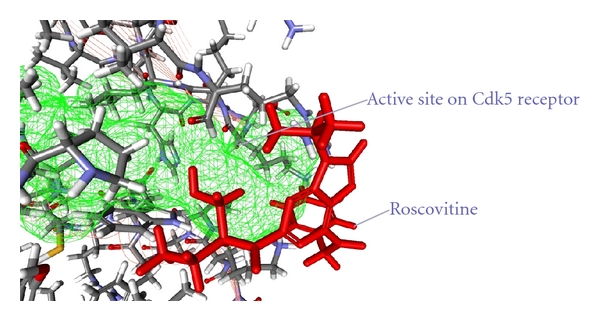
The docked conformation of Roscovitine with the receptor CDK5/p25 (PDB: 3O0G). The Roscovitine is indicated by red color while the most active site of the CDK5 receptor is shown with green color.

As from the docking with Discovery Studio 2.5 it is obvious that the most potent inhibitor of CDK5/p25 receptor as compared to the standard compounds, namely, Olomoucine and Roscovitine, is compound no. 6. The binding pattern of compound 6 is quite different from the other entire compounds, and it integrates tightly with the receptor in order to inhibit it (see Figures 4, 5, 6, 7, 8, 9, 10, 11, 12, 13).

## 8. Discussion and Conclusion

From the recent findings it has been suggested that CDK5 is an imperative contrivance which might prove to be a significant target for take care of type 2 diabetes mellitus. It has been shown from previous research study [7] that expression of p35 and the protein kinase activity of the p35/CDK5 complex in *β* cells are tightly regulated by changes in the extracellular concentration of glucose. It is known that the expression of genes essential for the function of *β* cells, such as insulin, PDX-1 (pancreatic and duodenal homeobox-1), amylin, and glucose transporter 2, is closely regulated by extracellular concentrations of glucose. This circumstance suggests that the p35/CDK5 protein kinase pathway may play an important role in *β*-cell function. Further it is apparent that inhibition of CDK5 under conditions of glucotoxicity preserves insulin gene expression by a mechanism that involves the restoration of the DNA binding and nuclear localization of PDX-1 and restoration of the nuclear levels of PDX-1 by inhibition of CDK5 occurs by preventing the translocation of PDX-1 from the nucleus to the cytoplasm [22].

It has been a long time since CDK5 was discovered. All-embracing studies have been conducted to elucidate the function of CDK5. Physiologically, CDK5 has been implicated in the regulation of neuronal development and synaptic transduction. Pathogenically, CDK5 has been accepted as a critical arbitrator in various neurodegenerative diseases, including Alzheimer's disease. Above and beyond the neuronal functions of CDK5, latest studies also advocate that CDK5 might regulate insulin secretion in pancreatic *β* cells. 

CDK5 is all over the place expressed, with high levels of expression in the central nervous system. It is multifunctional with predictable roles in signalling pathways and gene transcription, organisation of the cytoskeleton and focal adhesions, membrane dynamics, and function as well as cell metabolism [27]. More explicitly, in addition to its positive role in insulin secretion [9], CDK5 is also required for insulin-stimulated glucose uptake, upsetting translocation of GLUT4 [28, 29]. Therefore, aiming CDK5 to improve PPAR-*γ* activity and metabolic control is not without challenges. A more eye-catching approach is to coalesce this new consideration with our mushrooming admiration of the prospective selectivity and efficacy afforded by the CDK5/p25 inhibitors to endow with less adverse effects of Thiazolidinediones (TZDs). On the basis of these findings, it is obvious that CDK5 might be a constructive target for the treatment of diabetes. On the whole, several scientists and Choi and colleagues' work heralds a new epoch of drug discovery, in which PPAR*γ* activity could be rationally targeted to ameliorate diabetes and circumvent side effects.

## Figures and Tables

**Table 1:** IC_50_ (nM/*μ*M) of various CDK5 inhibitors.